# Association between the Healthy Eating Index-2015 and Developmental Disabilities in Children: A Cross-Sectional Analysis

**DOI:** 10.3390/brainsci13091353

**Published:** 2023-09-21

**Authors:** Jianxiong Gui, Lingman Wang, Ziyao Han, Ran Ding, Xiaoyue Yang, Jiaxin Yang, Hanyu Luo, Dishu Huang, Jie Liu, Li Jiang

**Affiliations:** National Clinical Research Center for Child Health and Disorders, Ministry of Education Key Laboratory of Child Development and Disorders, Chongqing Key Laboratory of Pediatrics, Department of Neurology, Children’s Hospital of Chongqing Medical University, Chongqing 400014, Chinayjx19950403@163.com (J.Y.); dr_hanyuluo@126.com (H.L.);

**Keywords:** developmental disabilities, children, dietary quality, HEI-2015, NHANES

## Abstract

Few studies have examined the association between dietary quality and the risk of developmental disabilities (DDs). This study aimed to investigate the association between dietary quality and the risk of DDs in US children aged 5 to 15. We employed data from the National Health and Nutrition Examination Survey (NHANES) 2003–2018. Multivariable logistic regression was used to evaluate the association between HEI-2015 score, HEI component score, and the likelihood of DDs. Restricted cubic splines (RCS) were utilized to investigate nonlinear links between HEI-2015 score and the likelihood of DDs. Interaction analysis was utilized to explore differences between subgroups. HEI-2015 score was negatively linked with the risk of DDs after adjusting covariates [odds ratio (OR) = 0.99; 95% confidence interval (CI) = (0.98, 1.00)]. HEI-2015 score was separated by quartile into Q1, Q2, Q3, and Q4. Q1 represents the lowest HEI scores, while Q4 represents the highest HEI scores. Children in the fourth quartile of the HEI-2015 exhibited a decreased prevalence of DDs compared to those in the first quartile [(OR = 0.69; 95% CI = (0.53, 0.89)]. The association between HEI-2015 score and the risk of DDs was modified by race/ethnicity. The higher HEI-2015 score was associated with a lower risk of DDs, suggesting that better dietary quality may reduce the risk of DDs in children.

## 1. Introduction

Developmental disabilities (DDs) are referred to as a cluster of life-long conditions characterized by difficulties in acquiring and executing specific intellectual, physical, linguistic, or social functions, based on the 10th edition of the International Classification of Diseases (ICD). DDs have an onset in childhood and mainly cover neurodevelopmental disorders with or without congenital abnormalities, unusual growth parameters, dysmorphic features, and atypical behavioral phenotypes [1,2]. Specific classifications of DDs include sensory impairments (hearing and vision loss), epilepsy or seizures, cerebral palsy, intellectual disability (ID), autism spectrum disorder (ASD), attention-deficit/hyperactivity disorder (ADHD), and other learning disorders [3]. Between 2009 and 2017, there was an elevated occurrence (from 16.2% to 17.8%) of DDs in children aged 3 to 17 years, as evidenced by data derived from the National Health Interview Survey (NHIS) [4]. From 2019 to 2021, there was an observed increase in the prevalence of diagnosed developmental disabilities in children aged 3 to 17 years, rising from 7.40% to 8.56% [5]. The precise mechanisms underlying the pathogenesis of DDs remain largely uncertain. Nevertheless, dietary choices and quality during childhood have the potential to impact both the brain’s structure and energy provision, thereby affecting the cognitive development of children [6]. According to a prospective birth cohort, poorer eating choices at preschool age, indicated by high-fat, high-salt, and high-sugar diets, are related to inferior verbal and cognitive abilities [7]. Another research study indicated a possible correlation between dietary exposure and the development of higher cognitive functions governed by the maturation of the frontal lobe in 7-year-old children [8].

Diet quality, which considers the overall effects and potential interactions of diet, can frequently be assessed using national dietary recommendations such as the Healthy Eating Index (HEI). HEI serves as a practical instrument to assess the quality of an individual’s dietary intake, with a higher HEI score indicative of greater adherence to the Dietary Guidelines for Americans (DGA) [9]. HEI focuses on high intake of total fruits, whole fruits, total vegetables, greens and beans, total protein foods, seafood, plant proteins, whole grains, dairy, and fatty acid, and moderate intakes of sodium, refined grains, saturated fats, and added sugar [10]. Previous studies examined the association between diet quality and health outcomes of various individuals using the HEI, such as the risk of depression, sleep disorders, and cardiovascular and cancer mortality [11,12,13]. The timeframe spanning childhood through early adolescence is a pivotal stage for both physical and cognitive maturation. Concurrently, changes in brain structure occur, marked by a decrement in gray matter and a concomitant increase in white matter [14], culminating in lasting effects on cognitive function [15]. Significantly, optimal nutritional intake and adequate dietary choices play a fundamental role in shaping neurodevelopmental outcomes among children. A study remarked that a diet comprised of refined sugar and saturated fat can raise the risk of ADHD, whereas a balanced diet rich in fruits and vegetables can mitigate this harmful effect [16]. The majority of children with ASD may benefit from a balanced nutritional and dietary intervention that improves their nutritional status, nonverbal intelligence quotient (IQ), and autism symptoms [17].

To date, there are few studies on the association between diet quality and DDs. To fill the knowledge gap, we aimed to explore the association between the HEI-2015 score and DDs in US children aged 5–15 from the NHANES 2003–2018.

## 2. Materials and Methods

### 2.1. Study Population

**Figure 1:**
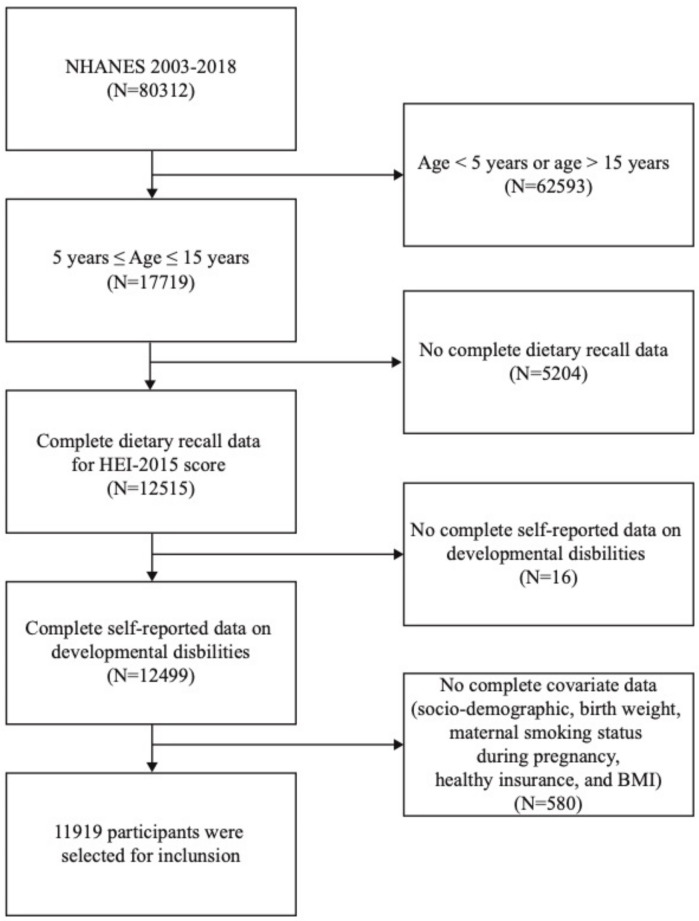
Flowchart outlining participant inclusion criteria.

The NHANES are performed by the US National Center for Health Statistics (NCHS) to evaluate the health and nutritional status of the US population using a complex multistage sampling design. On the official website (https://www.cdc.gov/nchs/nhanes/index.htm (accessed on 13 February 2023)), additional details about the NHANES protocol are provided. The sample for this study consisted of NHANES 2003–2018 participants aged 5 to 15 years old. All study data were obtained in the presence of the participant’s parents or guardians and with each participant’s written informed consent. The NHANES 2003–2018 included 17,719 participants aged 5 to 15. After applying the inclusion and exclusion criteria, 11,919 participants with complete data were selected for inclusion (Figure 1).

### 2.2. Assessment of HEI-2015 Score

Dietary data in NHANES were acquired from the program of the United States Department of Agriculture (USDA) titled “What We Eat in America” (WWEIA). We collected dietary information from 2-day recalls in the dietary intake interview component of the NHANES. The USDA Food Patterns Equivalence Database (FPED) was utilized for the calculation of food groups, nutrients, and calories.

Dietary quality was evaluated using HEI-2015, according to a continuous score from 0 to 100, following DGA recommendations. HEI-2015 contains thirteen food components, including nine adequacy and four moderate components. Six adequacy components (total fruits, whole fruits, total vegetables, greens and beans, total protein foods, seafood and plant proteins) were measured on a 0–5 range, while three adequacy components were measured on a 0–10 range (whole grains, dairy, fatty acid). The four moderate components (sodium, refined grains, saturated fats, and added sugar) are scored between 0 and 10 points. Detailed HEI-2015 scoring criteria can be found in Table S1. The total HEI-2015 score is obtained by summing the scores of the individual components, utilizing the “nhanesR” package. A higher HEI score indicates adherence to DGA-recommended dietary patterns, which is regarded as superior dietary quality.

### 2.3. Assessment of DDs

The information pertaining to a child’s diagnosis of DDs was ascertained through a process involving solicitation of responses from either the child’s parents or guardian, as it related to the following query: “Does the participant receive any form of specialized education or early intervention services?” Consequently, affirmative answers were designated as indicative of developmental disorders (DDs). Special education (SE) and early intervention (EI) are advantageous forms of support for children with developmental disabilities and/or delays, as they facilitate skill development and improve overall quality of life [18,19]. Similarly, former NHANES studies adopted the same self-report to ascertain DDs [20,21].

### 2.4. Covariables

Based on previous research on DDs in children and the complete information available from NHANES 2003–2018, covariables were identified [20,22]. These covariates included age, sex, race/ethnicity, body mass index (BMI), family poverty status, normal birth weight (5.5 lbs or more), maternal smoking status during pregnancy, and with/without health insurance coverage. Race/ethnicity were categories for non-Hispanic white, non-Hispanic black, Mexican American, and other races. Birth weight and maternal smoking during pregnancy were asked of children aged 0–15 years. BMI (kg/m^2^) was determined by dividing the participant’s weight by the square of their height. Poverty status was determined by the poverty income ratio of <1 (below the poverty threshold) versus ≥1 (reference).

### 2.5. Statistical Analysis

Initially, descriptive analyses presented continuous variables as weighted means (standard errors) and categorical variables as unweighted numbers (weighted percentages), if appropriate, utilizing the Student’s *t*-test or Chi-square.

Then, logistic regression was utilized to calculate prevalence odds ratios (ORs) and 95% confidence intervals (CIs) as a cross-sectional assessment of the relationship between the HEI-2015 score and DDs. The HEI-2015 score was modeled as a continuous and categorical variable. In the categorical variable, HEI-2015 score was separated by quartile into Q1, Q2, Q3, and Q4, with the lowest quartile, Q1, serving as the reference. The median of each category was used as a linear variable in the regression models to test for trend (*P*-trend). In model 1, the HEI-2015 score was the only independent variable, whereas age, sex, race/ethnicity, poverty status, birth weight, BMI, maternal smoking during pregnancy, and health insurance coverage status were considered in model 2. Meanwhile, we examined the nonlinear link between the HEI-2015 score and DDs using restricted cubic splines (RCS) in the logistic regression model. *p*-values for nonlinear trends were determined using Wald testing for RCS coefficients. Moreover, the HEI-2015 components were also employed as continuous variables in logistic regression to examine their association with DDs.

To determine whether the association varied among subgroups categorized by age (children aged 5–11 years and adolescents aged 12–15 years) [20,22], sex, race/ethnicity, poverty status, birth weight, maternal smoking status during pregnancy, health insurance coverage status, and BMI (<25, ≥25), interaction analysis was conducted by introducing an interaction term between the HEI-2015 score and subgroup status to the regression model.

We used the variance inflation factor to verify whether these covariates exhibit collinearity. And the correction of *p*-values for primary results was performed using the Benjamini and Hochberg FDR (BH) method as a sensitivity analysis, ensuring the robustness of our findings.

NHANES sample weights (wtdr2d) were utilized in R 4.2.1 for all statistical analyses, which properly accounted for the stratification and complexity of the NHANES sampling. All statistics were two-sided, and *p* < 0.05 was considered statistically significant.

## 3. Results

**Figure 2:**
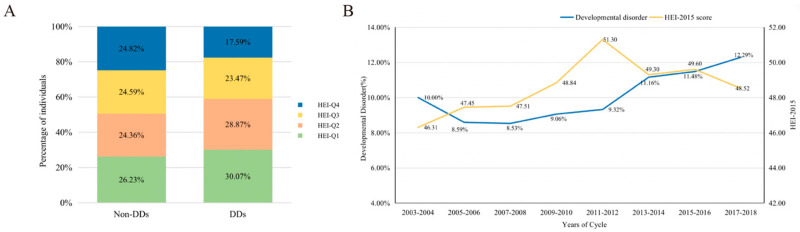
(**A**) Quartile distribution of HEI-2015 among DD and non-DD participants. (**B**) The prevalence of DDs and HEI-2015 score in NHANES cycles (2003–2018).

In total, 11,919 children aged 5–15 years from 2003–2018 NHANES were eligible for this study. Table 1 displays the participants’ demographic data grouped by DDs. Participants with DDs were more likely to be female, in poverty status, with low birth weight and experience maternal smoking during pregnancy, lower HEI-2015 score, and lower food composition score, including total vegetables, total fruit, whole fruits, seafood and plant proteins, and saturated fats. Participants with DDs showed a higher proportion of HEI scores in Q1 and Q2 and a lower proportion of HEI scores in Q3 and Q4 (Figure 2A). According to the HEI-2015 quartiles, the proportion of individuals with DDs in Q1 (17.05, 40.89], Q2 (40.89, 48.32], Q3 (48.32, 56.33], and Q4 (56.33, 82.53] was 10.97%, 11.56%, 10.07%, and 7.23%, respectively (Table S2).

In addition, we discovered that the prevalence of DDs increased from 2003 to 2018, reaching a maximum of 12.29% in the 2017–2018 cycle. The average HEI-2015 score rose first and subsequently declined, reaching a high of 51.30 in the 2011–2012 cycle (Figure 2B). The ratio of the average score of each component to the total score of the HEI-2015 is displayed in Figure S1.

**Figure 3:**
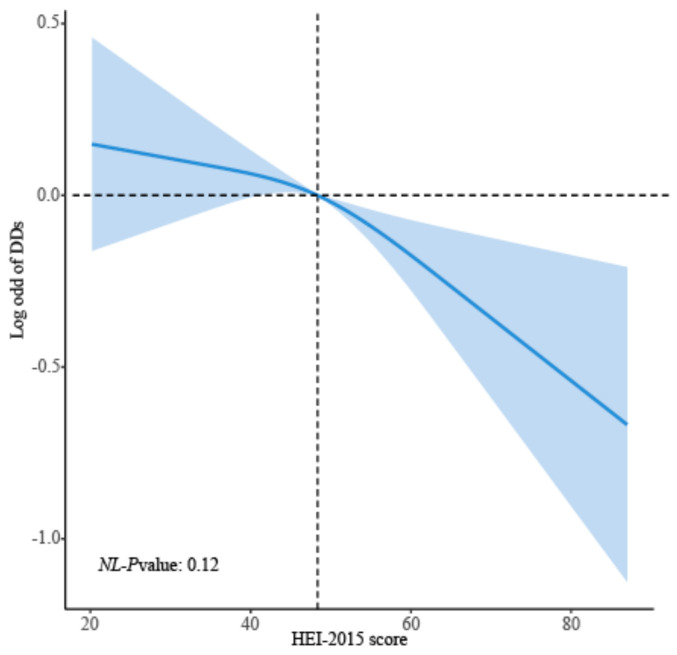
Smooth curve fitting of HEI-2015 score with DDs, adjusted for age, sex, race/ethnicity, poverty status, birth weight, BMI, maternal smoking during pregnancy, and health insurance coverage status; *p* for non-linearity (*NL*-*p* value) was used to evaluate the nonlinear relationship, with <0.05 indicating statistical significance.

When the HEI-2015 score was applied to the logistic regression analysis as a continuous variable, the HEI-2015 score was strongly related to decreased risk of DDs in model 1 (*p*-value < 0.001). After adjusting covariates in model 2, the relationship between the HEI-2015 score and the risk of DDs remained stable (*p*-value < 0.01). As the HEI-2015 score was designed to be categorical variables, compared with HEI-Q1, HEI-Q4 decreased the likelihood of DDs in model 1 and model 2 (HEI-Q4: (OR (95%CI): 0.63 (0.49, 0.82), 0.69 (0.53, 0.89), respectively; *p* for trend: <0.001, <0.01, respectively) (Table 2). Regression analysis applying RCS explored the potential nonlinear relationship between HEI-2015 and the likelihood of DDs. The risk of DDs tended to decrease linearly with increasing HEI-2015 scores, with no significant nonlinear turning points (*p* for non-linearity: 0.12) (Figure 3).

Multivariate logistic regression analysis of the components of the HEI-2015 revealed that the scores of total vegetables, total fruit, whole fruits, and saturated fats were substantially linked with the risk of DDs. The most closely linked components were total fruit (OR (95%CI): 0.93 (0.88, 0.98)), whole fruits (OR (95%CI): 0.94 (0.90, 0.98)), total vegetables (OR (95%CI): 0.94 (0.87, 1.00)), and saturated fats (OR (95%CI): 0.96 (0.93, 1.00)) (Table 3).

**Figure 4:**
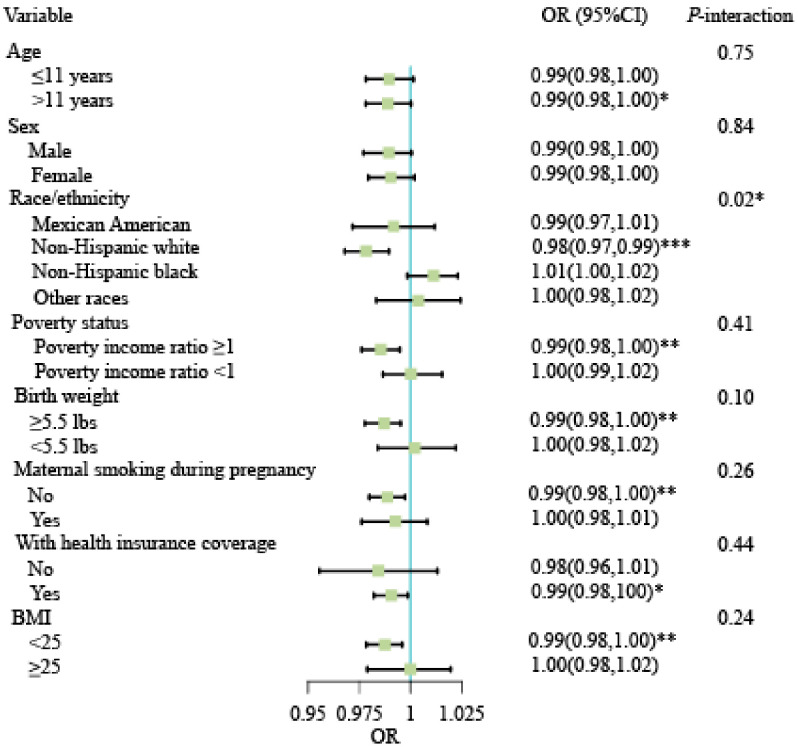
Subgroup analysis and interaction analysis of the association between the HEI-2015 score and DDs, adjusted for all covariables except for subgroup variables. *P*-interaction was used to evaluate the interaction, with <0.05 indicating statistical significance. * *p* < 0.05, ** *p* < 0.01, *** *p* < 0.001.

Subgroup analyses revealed that race/ethnicity was an effect modifier for the link between HEI-2015 score and the risk of DDs after adjusting for other covariates. The results demonstrated that the effect size of the link between race/ethnicity varied greatly. For race/ethnicity, the OR (95%CI) of Mexican American, non-Hispanic white, non-Hispanic black, and other races were 0.99 (0.97, 1.01), 0.98 (0.97, 0.99), 1.01 (1.00, 1.02), and 1.00 (0.98, 1.02), respectively, with *P* for interaction: 0.02 (Figure 4 and Table S3). There were no significant interactions for age, sex, poverty status, birth weight, maternal smoking status during pregnancy, health insurance coverage status, or BMI between HEI-2015 score and the risk of DDs. The variance inflation factors for the variables were less than 5, indicating the absence of collinearity (Figure S2). The sensitivity analysis showed the robustness of our findings (Table S4).

## 4. Discussion

This large cross-sectional study analyzing eight cycles of NHANES data assessed the relationship between the HEI-2015 score and the risk of DDs in US children aged 5 to 15 years. In light of the fact that the prevalence of DDs is increasing annually, it is imperative that we delve into potential preventive factors. We found that children with DDs had a lower HEI-2015 score and that participants in the fourth quartile of the HEI-2015 score had a lower prevalence of DDs. In addition, our results revealed that a higher HEI-2015 score was significantly related to a lower risk of DDs, which meant that a higher-quality diet was significantly associated with a lower risk of DDs. The link can be modified by race/ethnicity. Among the 13 components of the HEI-2015, the scores for total vegetables, total fruit, whole fruits, and saturated fats were most strongly negatively associated with the risk of DDs.

To the best of our knowledge, this was the first study to investigate the association between HEI-2015 scores and the risk of DDs in children. NHANES, as a large-scale epidemiological survey, provides a unique platform for examining the impact of diet quality on the general population. Leveraging NHANES data, numerous studies have assessed the association between the consumption of common foods, such as cereal, yogurt, orange juice, and raisins, and diet quality (assessed by HEI-2015 score) in US children [23,24,25,26]. Jun et al. studied the relationship between food insecurity and diet quality in US children through NHANES 2011–2016 [27]. However, limited studies have sought the association between dietary quality and the likelihood of DDs occurring in children. Some studies explored the link between diet quality and ASD, but there was no discernible difference from the control group, which may be due to the small sample size [28,29]. Furthermore, studies have connected healthier eating patterns to a lower self-reported risk of hearing impairment [30]. Indirect evidence linking diet quality to the risk of DDs has also been uncovered. Several prevalent childhood health problems (e.g., obesity, diabetes, metabolic syndrome, and inflammation) are intimately linked to diets. A series of effective dietary interventions prevent or reduce childhood obesity [31]. Additionally, the Mediterranean-type diet (MeDi) has been demonstrated to optimize glucose levels in patients with type 1 diabetes [32], and better dietary quality may hinder the development of metabolic syndrome in children and adolescents [33]. In contrast, an unhealthy diet, with a higher dietary inflammatory index, is also considered a risk factor for several chronic diseases [34]. Former investigations have indicated that DDs could be attributed to a plethora of risk factors, including but not limited to obesity, diabetes, metabolic syndrome, and heightened systemic inflammation [35,36,37,38]. For HEI-2015, a healthier diet quality means higher consumption of adequacy components (total fruits, whole fruits, total vegetables, greens and beans, total protein foods, seafood, plant proteins, whole grains, dairy, and fatty acid) and lower consumption of moderation components (sodium, refined grains, added sugars, and saturated fats). Our findings suggested that consuming more total fruits, whole fruits, and total vegetables, alongside fewer saturated fats, exhibited an inverse correlation with the likelihood of DDs.

There have been some researchers who have inquired into eating habits and the nutritional practices of children with DDs. In recent years, Kral et al. have summarized and described the feeding issues (such as prolonged mealtimes, fussy eating, food selectivity, etc.) and gastrointestinal dysfunction that may exist among individuals with ASD [39]. Children with ASD were more likely to have higher food preference, according to Curtin et al. [40]. As reported by Evans et al., children with ASD ingested a greater amount of juice, sweetened non-dairy drinks, and candy than those who were typically developing, but significantly fewer portions of vegetables [41]. From another perspective, our research found healthier eating habits were significantly associated with a lower risk of DDs. Furthermore, a 12-month randomized controlled experiment found that a comprehensive food program was safe and beneficial in increasing nutritional status, nonverbal intelligence quotient (IQ), communication, daily living abilities, and social skills, as well as reducing some autism symptoms [15]. A strong link between food and trajectory of development and recommended possible nutritional therapies for young autistic children was demonstrated in another study [42]. Therefore, the association between healthy diet and DDs is potentially bidirectional.

As per one study, a diet with fewer fruits and vegetables may be associated with more severe symptoms of inattention among children afflicted with ADHD [43]. Another research study showed that children with ID consumed fewer vegetables than children without ID, yet a causal relationship could not be established [44]. A class of flavonoids that were abundant in vegetables and fruits (e.g., anthocyanidins, found in berry fruits; flavonols, found in broccoli, onions, and leeks; flavanones, found in tomatoes and citrus fruit [45]) is believed to have favorable effects on the development of the central nervous system by protecting neurons from stress-induced damage, alleviating neuroinflammation, and enhancing cognitive performance [46]. A recent investigation has demonstrated that intestinal flora dysbiosis is directly associated with alterations in blood–brain barrier permeability and neuroinflammation, consequently impacting neurodevelopment [47]. Fruit fiber may alter gut function and intestinal flora dysbiosis [48]. Consistent with our findings, this indirect evidence reveals that higher vegetable and fruit intake may be related to a reduced risk of DDs. However, further high-quality basic and cohort studies are required to properly elucidate their causal links.

We discovered that race/ethnicity modified the association between HEI-2015 score and DD risk. Among non-Hispanic whites, the negative association between HEI-2015 score and the risk of DDs was more pronounced. We speculated that this may be due to differences in the prevalence of DDs among race/ethnicity groups and limited statistical efficacy in a small number of race/ethnicity subgroup subsamples. Similar race/ethnicity differences have been observed in previous studies exploring the relationship between dietary quality and cognitive decline and executive dysfunction, particularly among non-Hispanic whites [10,49]. Likewise, another NHANES study discovered a protective effect of the MeDi on the cognitive abilities of non-Hispanic whites, albeit not in other racial/ethnic groups [50].

In this study, we analyzed the association between HEI-2015 score and the risk of DDs in children aged 5 to 15 years in NHANES from 2003 to 2018. Our results can be generalized to a broader population of children by employing nationally representative data from a sophisticated stratified sample. Inevitably, our study included the following limitations. First of all, it is difficult to prove the causality between dietary quality and DDs in a cross-sectional study. Significantly more clinical cohort studies are required to verify the findings. Secondly, in accordance with the NHANES survey, children with ASD, ADHD, ID, motor development delays, hearing impairment, and speech impairment are eligible for special education or early intervention programs. Thus, the concept of DDs is inclusive of children with a variety of conditions. This restricted our ability to probe the relationship between diet quality and the incidence of specific developmental diseases, as well as the formulation of tailored dietary recommendations. Thirdly, the dietary information obtained through 2-day recalls in the dietary intake interview component of the NHANES was utilized for the calculation of HEI scores, which presents a limitation due to the possibility of memory decay and recall bias. Moreover, the assessment of DDs was based on self-report questionnaires rather than accurate clinical diagnoses. Therefore, further randomized controlled clinical trials are needed to validate our findings. Lastly, it is important to note that the inclusion of various age groups resulted in a disparity in the dietary requirements and eating behaviors observed between children aged 5–11 years and adolescents aged 12–15 years.

## 5. Conclusions

In conclusion, a higher HEI-2015 score was significantly associated with a lower risk of DDs. This inverse relationship was more obvious among non-Hispanic whites. This study also revealed that greater consumption of fruits and vegetables, as well as lower dietary intake of saturated fats, was negatively linked to the risk of DDs, offering guidance for more cohort studies with a lengthy follow-up in search of alternative therapies for children with DDs. Future research is warranted to fully ascertain the causal relationship and the underlying mechanism.

## Figures and Tables

**Table 1:** Characteristics of participants included in NHANES 2003–2018 analyses (*n* = 11,919) ^a^.

Variable	All (*n* = 11,919)	Non-DDs (*n* = 10,727)	DDs (*n* = 1192)	*p*-Value ^b^
Age	10.04 (0.04)	10.03 (0.05)	10.05 (0.13)	0.92
Sex				**<0.001**
Male	5964 (50.04)	5541 (50.53)	414 (35.17)	
Female	5955 (49.96)	5186 (49.47)	778 (64.83)	
Race/ethnicity				**0.01**
Mexican American	3011 (25.26)	2818 (14.76)	193 (12.14)	
Non-Hispanic white	3540 (29.7)	3148 (58.04)	392 (53.32)	
Non-Hispanic black	3164 (26.55)	2796 (13.37)	368 (16.31)	
Other races	2204 (18.49)	1965 (13.84)	239 (18.23)	
Poverty status				**<0.001**
No	8203 (68.82)	7482 (78.40)	721 (65.79)	
Yes	3716 (31.18)	3245 (21.60)	471 (34.21)	
Birth weight				**<0.001**
≥5.5 lbs	10,083 (84.6)	9140 (86.38)	943 (79.84)	
<5.5 lbs	1836 (15.4)	1587 (13.62)	249 (20.16)	
Maternal smoking during pregnancy				**<0.001**
No	5697 (87.51)	5157 (87.43)	540 (77.89)	
Yes	813 (12.49)	661 (12.57)	152 (22.11)	
Health insurance coverage				**0.04**
No	1203 (10.09)	1128 (8.09)	75 (5.76)	
Yes	10,716 (89.91)	9599 (91.91)	1117 (94.24)	
BMI	19.88 (0.10)	19.83 (0.11)	20.30 (0.23)	**0.05**
HEI score	48.79 (0.26)	48.96 (0.26)	47.24 (0.51)	**<0.01**
Total vegetables	2.26 (0.02)	2.28 (0.02)	2.14 (0.06)	**0.03**
Greens and beans	1.13 (0.03)	1.14 (0.03)	1.06 (0.08)	0.34
Total fruit	2.76 (0.04)	2.80 (0.04)	2.47 (0.10)	**0.001**
Whole fruits	2.68 (0.05)	2.72 (0.05)	2.32 (0.10)	**<0.001**
Whole grains	2.57 (0.05)	2.58 (0.05)	2.49 (0.13)	0.55
Dairy	7.35 (0.05)	7.34 (0.05)	7.38 (0.14)	0.82
Total protein foods	3.89 (0.02)	3.89 (0.02)	3.90 (0.06)	0.78
Seafood and plant proteins	2.01 (0.04)	2.03 (0.04)	1.83 (0.09)	**0.03**
Fatty acid	3.55 (0.05)	3.57 (0.05)	3.38 (0.13)	0.14
Sodium	4.75 (0.05)	4.76 (0.05)	4.68 (0.13)	0.57
Refined grains	4.77 (0.06)	4.76 (0.06)	4.83 (0.12)	0.62
Saturated fats	5.29 (0.06)	5.33 (0.06)	4.99 (0.13)	**0.02**
Added sugars	5.77 (0.06)	5.78 (0.06)	5.77 (0.13)	0.98

^a^ Continuous data were displayed as weighted means (standard errors), while categorical variables were exhibited as unweighted numbers (weighted percentages). BMI, body mass index; NHANES, the National Health and Nutrition Examination Survey; HEI, Healthy Eating Index. ^b^ *p*-values were calculated using Chi-square tests for categorical variables and Student’s *t*-test for continuous variables.

**Table 2:** Association between the HEI-2015 score and DDs (NHANES 2003–2018).

Variable	Model 1 ^a^		Model 2 ^b^	
	OR (95%CI)	*p*-Value/ *P*-Trend ^c^	OR (95%CI)	*p*-Value/ *P*-Trend ^c^
HEI-2015 score (continuous)	**0.99 (0.98, 0.99)**	**0.001**	**0.99 (0.98, 1.00)**	**0.01**
Quartile of HEI-2015		**<0.001**		**<0.01**
Q1 [12.66, 40.91]	Ref		Ref	
Q2 (40.91, 48.34]	1.06 (0.84, 1.33)		1.07 (0.84, 1.36)	
Q3 (48.34, 56.39]	0.91 (0.70, 1.18)		0.95 (0.73, 1.25)	
Q4 (56.39, 95.65]	**0.63 (0.49, 0.82)**		**0.69 (0.53, 0.89)**	

^a^ Model 1 was a crude model with no adjusted covariates. ^b^ Model 2 adjusted for age, sex, race/ethnicity, poverty status, birth weight, BMI, maternal smoking during pregnancy, and health insurance coverage status. ^c^ *P*-trend based on a variable containing the median value for each quartile.

**Table 3:** Association between the HEI-2015 components and DDs (NHANES 2003–2018).

HEI-2015 Components	Model 1 ^a^		Model 2 ^b^	
	OR (95%CI)	*p*-Value	OR (95%CI)	*p*-Value
Total vegetables	**0.93 (0.86, 0.99)**	**0.03**	**0.94 (0.87, 1.00)**	**0.05**
Greens and beans	0.97 (0.92, 1.03)	0.35	0.99 (0.94, 1.05)	0.77
Total fruit	**0.91 (0.87, 0.96)**	**0.001**	**0.93 (0.88, 0.98)**	**<0.01**
Whole fruits	**0.92 (0.88, 0.96)**	**<0.001**	**0.94 (0.90, 0.98)**	**<0.01**
Whole grains	0.99 (0.95, 1.03)	0.55	1.00 (0.96, 1.04)	0.82
Dairy	1.00 (0.97, 1.04)	0.82	1.00 (0.97, 1.04)	0.83
Total protein foods	1.01 (0.94, 1.09)	0.78	0.99 (0.92, 1.06)	0.76
Seafood and plant proteins	**0.95 (0.91, 1.00)**	**0.03**	0.96 (0.92, 1.01)	0.09
Fatty acid	0.98 (0.95, 1.01)	0.15	0.98 (0.95, 1.01)	0.26
Sodium	0.99 (0.96, 1.02)	0.57	1.00 (0.97, 1.03)	0.88
Refined grains	1.01 (0.98, 1.03)	0.62	1.00 (0.97, 1.03)	0.99
Saturated fats	**0.96 (0.93, 0.99)**	**0.02**	**0.96 (0.93, 1.00)**	**0.03**
Added sugars	1.00 (0.97, 1.03)	0.98	1.01 (0.97, 1.04)	0.68

^a^ Model 1 was a crude model with no adjusted covariates. ^b^ Model 2 adjusted for age, sex, race/ethnicity, poverty status, birth weight, BMI, maternal smoking during pregnancy, and health insurance coverage status.

## Data Availability

Publicly available datasets were analyzed in this study.

## Supplementary Materials

The following supporting information can be downloaded at https://www.mdpi.com/article/10.3390/brainsci13091353/s1, Figure S1: the ratio of the average score of each component to the total score of the HEI-2015; Figure S2: the variance inflation factor of variables; Figure S3: smooth curve fitting of HEI-2015 score with DDs by race/ethnicity; Table S1: HEI–2015 Components and Scoring Standards ^a^; Table S2: characteristics of participants included in NHANES 2003–2018 analyses by HEI-2015 quartile (n = 11,919) a; Table S3: subgroup analysis and interaction analysis of the association between the HEI-2015 score and DDs (NHANES 2003–2018); Table S4: correction of *p*-values for primary results using the Benjamini and Hochberg FDR (BH) method.
