# Supplementary material for: Association between the Healthy Eating Index-2015 and Developmental Disabilities in Children: A Cross-Sectional Analysis
Source: Brain Sci. 2023 Sep 21;13(9):1353. doi: 10.3390/brainsci13091353 (PMC10526872; doi:10.3390/brainsci13091353)
Supplement: Supplementary file 1 [file brainsci-13-01353-s001.zip › brainsci-2504900-supplementary.pdf]

Supplementary materials

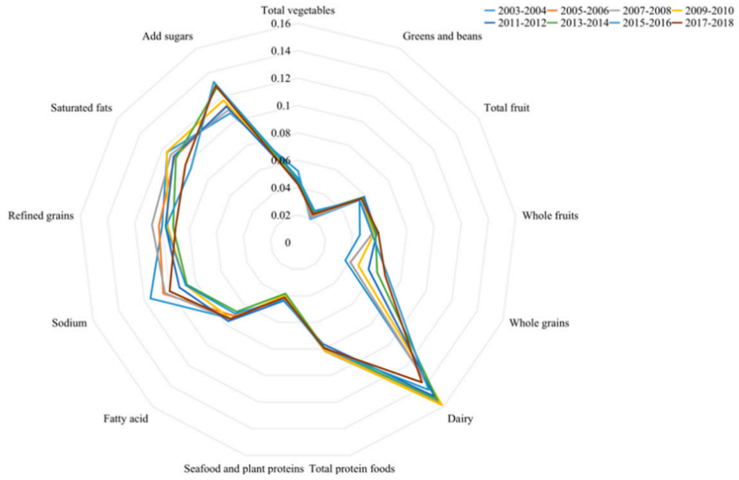

Figure. S1 The ratio of the average score of each component to the total score of the HEI-2015.

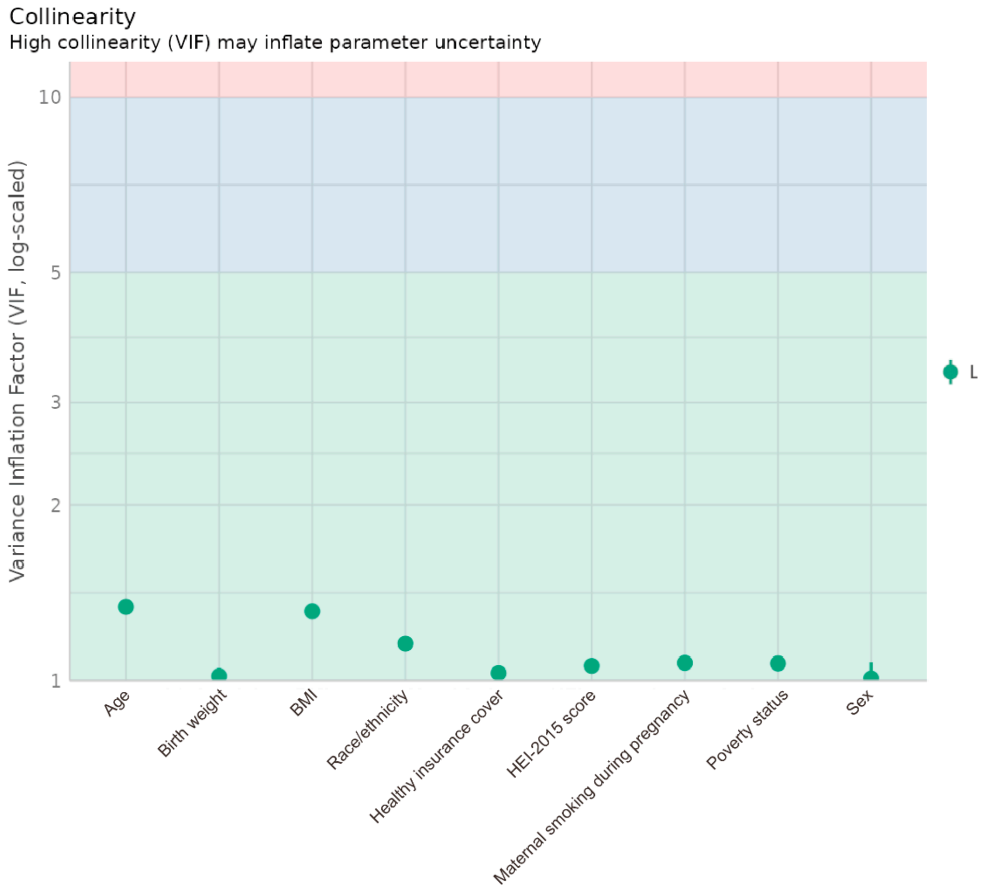

Figure. S2 The variance inflation factor of variables.

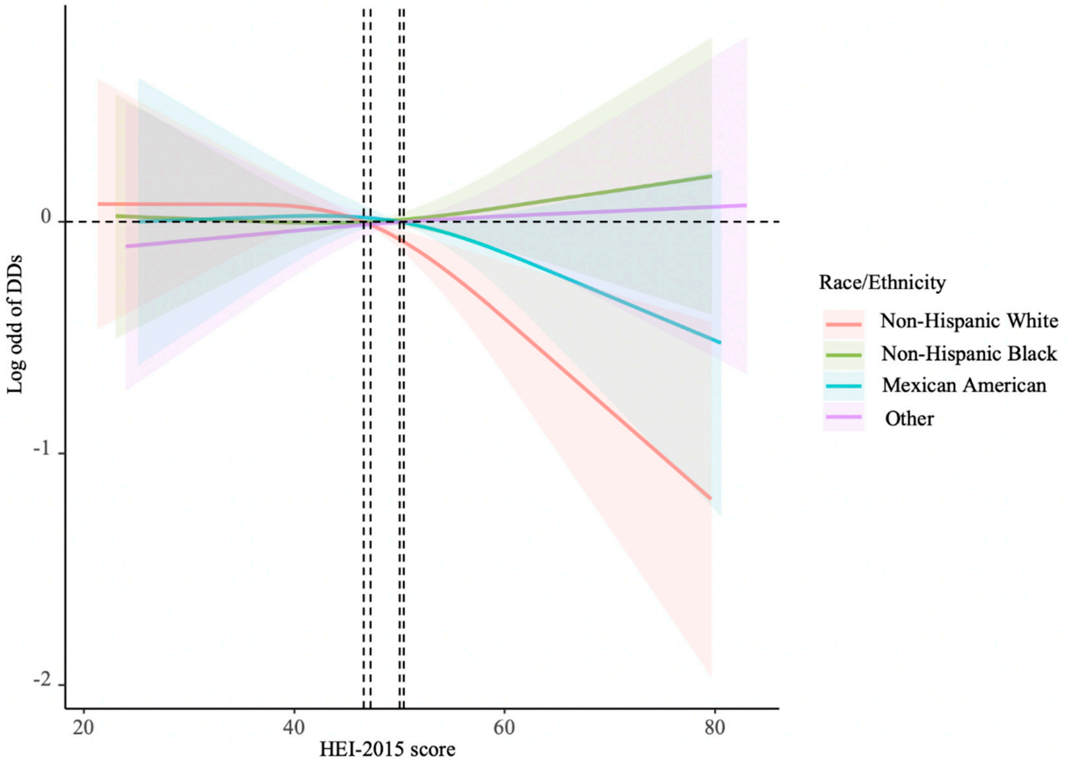

Figure. S3 Smooth curve fitting of HEI-2015 score with DDs by race/ethnicity.

Table. S1 HEI–2015 Components and Scoring Standards[a]

HEI-2015 Components	Range of Points	Minimum Scoring Standard	Maximum Scoring Standard
**Adequacy Components** (higher score indicates higher consumption)			
Total Fruits	0-5	0	0.8 cup equiv. /1000 kcal
Whole Fruits	0-5	0	0.4 cup equiv./1000 kcal
Total Vegetables	0-5	0	1.1 cup equiv. /1000 kcal
Greens and Beans	0-5	0	0.2 cup equiv. /1000 kcal
Total Protein Foods	0-5	0	2.5 oz equiv./1000 kcal
Seafood and Plant Proteins	0-5	0	0.8 oz equiv./1000 kcal
Dairy	0-10	0	1.3 cup equiv./1000 kcal
Whole Grains	0-10	0	1.5 oz equiv. /1000 kcal
Fatty Acids[b]	0-10	(PUFAs + MUFAs)/SFAs ≤1.2	(PUFAs + MUFAs)/SFAs ≥2.5

**Moderation Components** (higher score indicates lower consumption)			
Refined Grains	0-10	4.3 oz equiv./1000 kcal	1.8 oz equiv. /1000 kcal
Sodium	0-10	2.0 grams /1000 kcal	1.1 grams/1000 kcal
Added Sugars	0-10	26% of energy	6.5% of energy
Saturated Fats	0-10	16% of energy	8% of energy

[a]Intakes between the minimum and maximum standards are scored proportionately.

[b]Ratios of polyunsaturated and monounsaturated fatty acids (PUFAs and MUFAs) to saturated fatty acids (SFAs).

Table. S2 Characteristics of participants included in NHANES 2003-2018 analyses by HEI-2015 quartile(n=11919) [a].

**Variable**	**All**	**HEI-Q1**	**HEI-Q2**	**HEI-Q3**	**HEI-Q4**	***P*-value[b]**
**Age**	10.04(0.04)	10.67(0.08)	10.24(0.09)	9.69(0.09)	9.50(0.10)	**< 0.001**
**BMI**	19.88(0.10)	20.53(0.17)	20.04(0.15)	19.42(0.14)	19.50(0.16)	**< 0.001**
**Sex**						**0.05**
Male	5964(50.04)	1573(53.11)	1503(51.33)	1466(51.84)	1422(47.64)	
Female	5955(49.96)	1407(46.89)	1477(48.67)	1514(48.16)	1557(52.36)	
**Race/ethnicity**						**< 0.001**
Mexican American	3011(25.26)	587(10.77)	751(14.16)	774(15.09)	899(18.15)	
Non-Hispanic white	3540(29.7)	1086(64.02)	868(57.61)	824(55.06)	762(53.26)	
Non-Hispanic black	3164(26.55)	844(13.14)	864(15.01)	797(14.64)	659(11.90)	
Other races	2204(18.49)	463(12.07)	497(13.23)	585(15.21)	659(16.69)	
**Poverty status**						0.59
No	8203(68.82)	2095(78.25)	2015(75.80)	2056(77.61)	2037(76.84)	
Yes	3716(31.18)	885(21.75)	965(24.20)	924(22.39)	942(23.16)	
**Birth weight**						0.90
≥5.5 lbs	10083(84.6)	2511(85.19)	2522(86.28)	2516(85.68)	2534(85.81)	
<5.5 lbs	1836(15.4)	469(14.81)	458(13.72)	464(14.32)	445(14.19)	

**Maternal smoking during pregnancy**						**< 0.01**
No	5697(87.51)	1355(83.33)	1403(83.42)	1434(87.16)	1505(91.82)	
Yes	813(12.49)	273(16.67)	224(16.58)	193(12.84)	123(8.18)	
**With health insurance coverage**						0.38
No	1203(10.09)	263(7.16)	288(7.17)	314(8.49)	338(8.62)	
Yes	10716(89.91)	2717(92.84)	2692(92.83)	2666(91.51)	2641(91.38)	
**DDs**						**<0.01**
No	10727(90)	2635(89.03)	2660(88.44)	2690(89.93)	2742(92.77)	
Yes	1192(10)	345(10.97)	320(11.56)	290(10.07)	237(7.23)	

[a]Continuous data were displayed as weighted means (standard errors), while categorical variables were exhibited as unweighted numbers (weighted percentages). BMI, body mass index; NHANES, the national health and nutrition examination survey; HEI, Healthy Eating Index.

[b]*P*-values were calculated using Chi-square tests for categorical variables and Student's t-test for continuous variables.

Table. S3 Subgroup analysis and interaction analysis of the association between the HEI-2015 score and DDs (NHANES 2003-2018).

Variable	OR (95%CI)[a]	*P*-value	*P*-interaction[b]
**Age**			0.71
≤11 years	0.99(0.98,1.00)	0.06	
>11 years	0.99(0.98,1.00)	0.10	
**Sex**			0.84
Male	0.99(0.98,1.00)	0.06	
Female	0.99(0.98,1.00)	0.10	
**Race/ethnicity**			**0.02**
Mexican American	0.99(0.97,1.01)	0.41	
Non-Hispanic white	0.98(0.97,0.99)	<0.001	
Non-Hispanic black	1.01(1.00,1.02)	0.09	
Other races	1.00(0.98,1.02)	0.72	
**Poverty status**			0.41

No	0.99(0.98,1.00)	<0.01	
Yes	1.00(0.99,1.02)	0.91	
**Birth weight**			0.10
≥5.5 lbs	0.99(0.98,1.00)	<0.01	
<5.5 lbs	1.00(0.98,1.02)	0.78	
**Maternal smoking during pregnancy**			0.17
No	0.99(0.98,1.00)	0.07	
Yes	1.00(0.98,1.02)	0.79	
**With health insurance coverage**			0.44
No	0.98(0.96,1.01)	0.27	
Yes	0.99(0.98,100)	0.02	
**BMI**			0.24
<25	0.99(0.98,1.00)	<0.01	
≥25	1.00(0.98,1.02)	0.93	

[a]Adjust for all covariables except for subgroup variables.

[b]*P*-interaction was used to evaluate the interaction, with <0.05 indicating statistical significance.

Table. S4 Correction of p-values for primary results using the Benjamini and Hochberg FDR (BH) method.

**Variable**	**Model 1[a]**			**Model 2[b]**		
	**OR (95%CI)**	***P*-value**	**Adjust *P*-value**	**OR (95%CI)**	***P*-value**	**Adjust *P*-value**
**Whole fruits**	0.92(0.88,0.96)	**<0.001**	**0.001**	0.94(0.90,0.98)	**<0.01**	**0.04**
**Total fruit**	0.91(0.87,0.96)	**0.001**	**<0.01**	0.93(0.88,0.98)	**0.01**	**0.04**
**HEI2015-score**	0.99(0.98,0.99)	**0.001**	**<0.01**	0.99(0.98,1.00)	**0.01**	**0.04**
**Saturated fats**	0.96(0.93,0.99)	**0.02**	0.06	0.96(0.93,1.00)	**0.03**	0.09
**Total vegetables**	0.93(0.86,0.99)	**0.03**	0.07	0.94 (0.87,1.00)	**0.05**	0.15
**Seafood and plant proteins**	0.95(0.91,1.00)	**0.03**	0.07	0.96(0.92,1.01)	0.09	0.21
**Fatty acid**	0.98(0.95,1.01)	0.15	0.30	0.98(0.95,1.01)	0.26	0.52
**Add sugars**	1.00(0.97,1.03)	0.98	0.98	1.01(0.97,1.04)	0.68	0.95
**Total protein foods**	1.01(0.94,1.09)	0.78	0.88	0.99(0.92,1.06)	0.76	0.95
**Greens and beans**	0.97(0.92,1.03)	0.35	0.61	0.99(0.94,1.05)	0.77	0.95
**Whole grains**	0.99(0.95,1.03)	0.55	0.78	1.00(0.96,1.04)	0.82	0.95
**Dairy**	1.00(0.97,1.04)	0.82	0.88	1.00(0.97,1.04)	0.83	0.95
**Sodium**	0.99(0.96,1.02)	0.57	0.78	1.00(0.97,1.03)	0.88	0.95
**Refined grains**	1.01(0.98,1.03)	0.62	0.78	1.00(0.97,1.03)	0.99	0.99

[a]Model 1 was a crude model with no adjusted covariates.

[b]Model 2 adjusted for age, sex, race/ethnicity, poverty status, birth weight, BMI, maternal smoking during pregnancy, and health insurance coverage status.
